# Creatinine recovery from bovine urine under the effect of different times and temperatures of storage

**DOI:** 10.1371/journal.pone.0282145

**Published:** 2023-03-31

**Authors:** Jarbas Miguel da Silva Júnior, Luciana Navajas Rennó, Edenio Detmann, Sebastião de Campos Valadares Filho, João Paulo Pacheco Rodrigues

**Affiliations:** 1 Department of Animal Science of Sertão, Federal University of Sergipe, Nossa Senhora da Glória, Sergipe, Brazil; 2 Department of Animal Science, Federal University of Viçosa, Viçosa, Minas Gerais, Brazil; 3 Department of Animal Nutrition and Management, Swedish University of Agricultural Sciences, Uppsala, Sweden; 4 Department of Animal Science, Federal Rural University of Rio de Janeiro, Seropédica, Rio de Janeiro, Brazil; University of Jeddah, SAUDI ARABIA

## Abstract

Creatinine is a urinary marker used widely in ruminant’s experimental trials. However, despite its great importance no data were found in the literature about the best way to store bovine urine samples. In the sheep urine, was observed an increase in the urinary concentration of creatinine when it was stored acidified (pH 2.5 to 3.5) at a temperature of 28 to 39 °C for 150 days of storage. Nevertheless, urine should be stored acidified (pH below 3) to avoid purine derivative degradation, So, aimed to evaluate creatinine recovery in bovine urine as a function of storage time and temperature. A total of 25 animals’ urine (10 Nellore cattle and 15 Holstein cattle) were collected. The urine (40 mL) was diluted in 160 mL of distilled water and its pH was corrected to a value lower than 3 using sulfuric acid drops. A sample of the diluted urine was analyzed to obtain the creatinine concentration reference value on the collection day. The remaining urine was fractionated and preserved at room temperature, cooled (4 °C) or frozen (-20 °C and -40 °C). In the urine of five Holstein cattle was added creatine solutions (20, 40 and 60 mg/dL) to evaluate the creatine to creatinine conservation. These urine samples were analyzed on different days after collection (1, 3, 7, 15, 30 and 45 days). The urine without any added creatine was analyzed on Days 1, 3, 7, 10, 15, 30, 45, 60, 90, 120, and 150 of storage. The addition of creatine in the urine caused an increase in the creatinine concentration (P < 0.05) after 30 days of storage at room temperature and under refrigeration (4 °C). In frozen samples, there was no change in creatinine concentration (P > 0.05). However, creatinine recovery was constant (P > 0.05) until day 15 of storage, regardless of the temperature used, when creatine was not added. After 30 days of storage, an effect of time and/or temperature was observed on creatinine recovery (P < 0.05). Urine samples can be stored at any temperature for up to 15 days after collection to estimate the creatinine concentration. Samples that need storage times longer than 15 days should be frozen (at -20 °C and -40 °C) to avoid creatinine concentration variation.

## 1. Introduction

Urine is a biological fluid formed from blood clearance; it is easy to obtain and consists of the main route of excretion of nitrogenous compounds and purine derivatives in cattle [1]. These factors give urine great importance in animal experimentation, since these metabolites that are excreted through the urine serve as parameters to evaluate the digestion of dietary components, as well as metabolism dynamics, mainly nitrogen.

However, to obtain significant urine sample, it is necessary to perform urine collection for at least five days [2], which makes it a laborious experimental technique that is uncomfortable for the animal, even more when the animals are kept on pasture. Aiming to reduce this time, creatinine has been evaluated and validated as an internal marker of urinary volume [3–6]. Creatinine is a molecule formed by nonenzymatic water removal from creatine phosphate, which is an important energy reserve in muscle tissue metabolism [7–9]. Creatine phosphate degradation occurs relatively constantly and spontaneously. Approximately 2% of the body’s creatine pool is converted to creatinine daily [10]. Creatinine can be found in blood and urine. However, the creatinine present in the blood is no longer used by the animal; thus, it is filtered and excreted by the kidneys through the urine [8].

However, despite the great importance of knowing the concentration of creatinine in urine samples from cattle, no data were found in the literature about the best way to store urine samples. Which is a huge problem in animal science, once the increase or decrease in its concentration can lead to errors in the experimental data. Van Niekerk et al. [11], when evaluating creatinine recovery in the urine of sheep, observed that there was creatinine degradation at its normal pH (between 8.4 and 8.7) when stored at a temperature between 27 and 30 degrees Celsius (°C). The authors also observed an increase in the urinary concentration of creatinine when it was stored acidified (pH 2.5 to 3.5) at a temperature of 28 to 39 °C for 150 days of storage. Nevertheless, urine should be stored acidified (pH below 3) to avoid purine derivative degradation [2]. Which is a problem, once creatine if present in the acid urine it could be converted to creatinine, increasing the creatinine concentration [8, 9] what can lead to experimental errors.

Thus, we hypothesized that cattle urine with a pH below 3 and frozen (-20 °C and -40 °C) would not undergo changes in the urine creatinine concentration over 150 days of storage. The objective was to evaluate creatinine recovery as a function of different storage times and temperatures.

## 2. Materials and methods

### 2.1. Ethical considerations

The experiment was approved by the Ethics Committee on the Use of Production Animals—CEUAP of the Federal University of Viçosa (UFV), according to protocol No. 24/2016. The experiment was carried out at the Animal Science Department of UFV.

### 2.2. Urine samples and management

Urine samples from 25 animals were used; ten collected from beef cattle (five male steers and five female Nellore heifers) and fifteen from dairy cattle (five primiparous cows and ten multiparous Holstein cows). The cattle were chosen randomly from the animals that belong to the animal science department of UFV. So, there were chosen animals with 450 ± 48 kg of body weight and more than 16 months old.

Sampling was performed during spontaneous urination (*spot* urine collection) by collecting 40 milliliters (mL) of urine per animal via *spot* urine collection, following the recommendations of Chen and Gomes [2]. The urine samples were diluted in 160 mL of distilled water to avoid precipitation of uric acid and had its pH adjusted to values below 3 to avoid microbial degradation of purine and creatinine derivatives.

Immediately after sampling, dilution, pH correction and homogenization, an aliquot was analyzed to determine the creatinine concentration on the collection day, which was established at day zero. The remaining urine samples were fractionated; after homogenization, the other aliquots were stored in 2-mL Eppendorf tubes and stored in Eppendorf boxes with a capacity of 100 units.

At total 20 urine samples (10 from Nellore and 10 from Holstein cattle) were choose randomly to be used to evaluate creatinine recovery due the time and temperature storage. The last five urine from Holstein cattle was used to evaluate the creatine to creatinine conversion as a function of storage time and temperature. To do so, 0.2 mL of creatine solution was added to 1.8 mL of urine at a pH below 3 in Eppendorf tubes. To prepare the creatine solution, a SIGMA^®^ (São Paulo, Brazil) analysis standard was used with 99.5% purity. The aliquots were then stored, after homogenization, in Eppendorf boxes with a capacity for 100 units, one aliquot of urine diluted without the addition of creatine solution and three with the addition of creatine solution at 20, 40 and 60 milligram per deciliter (mg/dL).

### 2.3. Urine storage time and temperature

Urine with the added creatine solution was analyzed on Days 1, 3, 7, 15, 30 and 45 after collection. Urine without creatine solution addition was analyzed on Days 1, 3, 7, 10, 15, 30, 45, 60, 90, 120 and 150 of storage after collection.

Both urines (with or without added creatine solution) were stored at room temperature, kept under refrigeration at 4 °C, and frozen at -20 °C and -40 °C, totaling four storage temperatures.

Were used a regular room for urine storage in the laboratory of the Animals Science Department of UFV. The ambient temperature of the room where the urine related to this variable was checked and recorded every day at 4:00 pm, the time when the maximum ambient temperature was already reached. To measure the ambient temperature, a meteorological thermometer was used with maximum and minimum temperature measurements.

### 2.4. Creatinine analysis

To determine the creatinine concentration, the colorimetric kinetic method (K067) from the Bioclin^®^ kit (Belo Horizonte, Brazil) was used. To do so, we used an automated biochemistry analyzer (Mindray, model: BS200E, Belo Horizonte, Brazil) with a wavelength of 510 nanometers (nm) reading (490–520 nm) at the Laboratory of Animal Physiology and Reproduction of the Animal Science Department of the UFV.

### 2.5. Relative creatinine and statistical analysis

The experiment was conducted as a completely randomized design with 20 urine samples (10 from Nellore and 10 from Holstein cattle) used to evaluate the creatinine concentration recovery; and 5 urine samples from Holstein cattle with creatine added to evaluate the creatine to creatinine conversion due the time and temperature storage.

The creatinine concentration on day zero served as a reference for the stored samples. The value of the creatinine concentration (mg/dL) on the day of the analysis was divided by the reference value, thus obtaining the relative creatinine value. This allows us to observe whether the creatinine concentration increased or decreased in urine samples due to time and storage temperature.

To assess the conversion of creatine into creatinine, the statistical analysis system (SAS) 9.4 regression (REG) procedure was used, where the linear regression of added and retrieved values was estimated, with the regressor parameter (β1) interpreted as a test of creatine to creatinine conversion. A 5% alpha was adopted as the critical level of probability for type I error.

To evaluate creatinine recovery over time under the effect of storage temperature, the statistical procedures of the SAS were used. The MIXED procedures were used, adopting a 5% alpha as the critical level of probability of type I error, with the storage time and temperatures as fixed effects, and the animals as random effects.

## 3. Results and discussion

The average ambient temperature observed in the sample storage room was 20.6 ± 1.1 °C for a minimum and maximum of 26.4 ± 1.1 °C.

**Fig 1:**
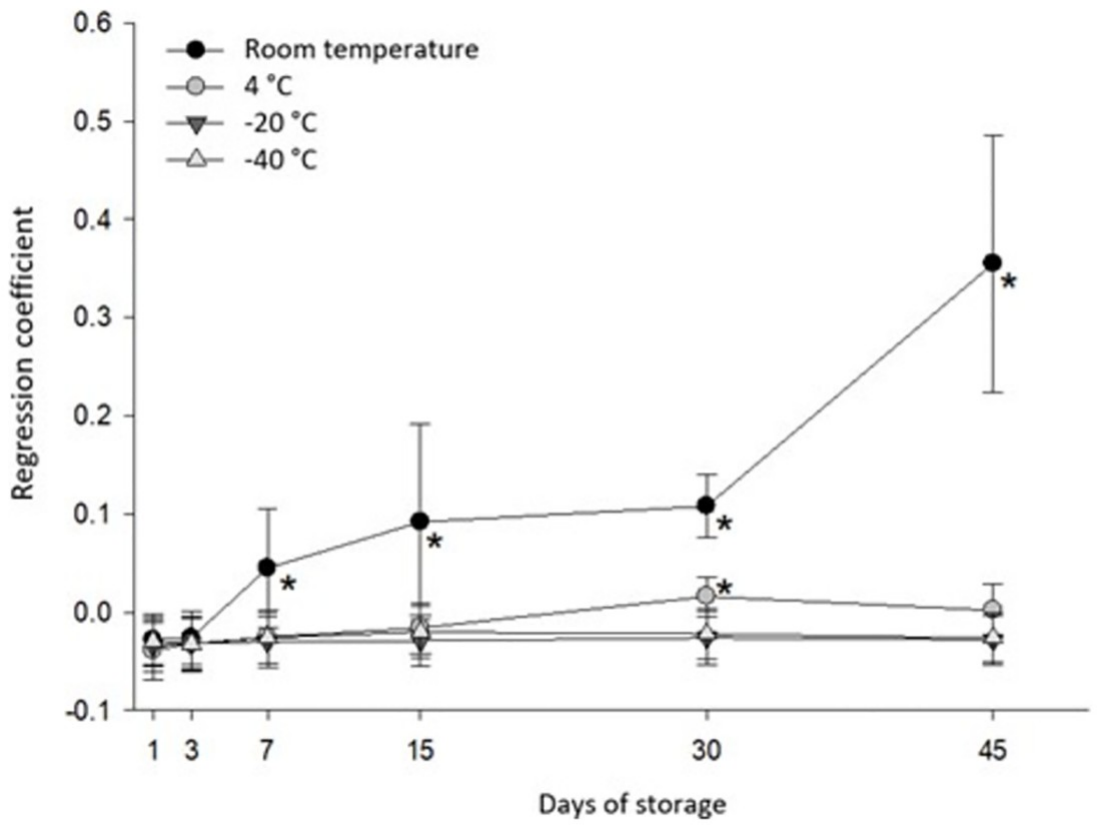
Coefficients of linear regression between creatinine values analyzed as a function of added creatine, according to days of storage at different temperatures.

Adding a creatine solution (Fig 1) to the cattle urine showed that as the days of storage passed, there was an increase (P < 0.05) in creatinine concentration when stored at room temperature (24.3 °C) and refrigerated (4 °C) after 30 days. At freezing temperatures (-20 °C and -40 °C), there was no change in creatinine concentration (P > 0.05).

**Fig 2:**
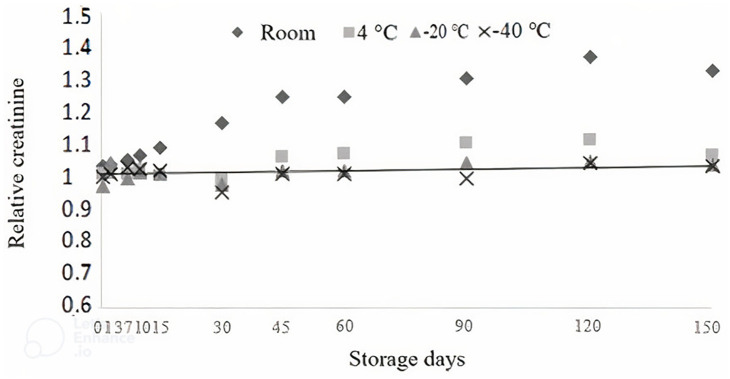
Relative creatinine concentration in cattle urine as a function of time (days) and temperature storage.

The creatinine increased since the 30th day of storage in the experiment without creatine addition to urine (Table 1 and Fig 2). In the experiment, creatine inclusion occurred earlier, on the 7th day of storage. On the 7th day of storage, for each milligram of creatine added, approximately 5% was converted into creatinine; at 45 days of storage, this conversion was approximately 35%, showing that the higher the concentration of creatine in the urine, the greater and faster its conversion to creatinine. Thus, we confirmed the hypothesis that urine preserved without freezing, especially at room temperature, can increase the conversion of creatine into creatinine in acidified urine (pH < 3).

**Table 1:** Means, significance level (P—values) and standard error of the mean (SEM) obtained for relative creatinine as a function of time (days) and storage temperature in urine samples from cattle.

Days	Temperature				P–Value^a^
	Room	4 °C	-20 °C	-40 °C	
1	1.037	1.015	0.975	1.001	0.376
3	1.039	1.015	1.045	1.012	0.759
7	1.054	1.013	0.998	1.003	0.441
10	1.067	1.027	1.017	1.028	0.527
15	1.092	1.009	1.011	1.021	0.067
30	1.168	0.995	0.980	0.958	<0.001
45	1.248	1.065	1.022	1.014	<0.001
60	1.250	1.074	1.023	1.015	<0.001
90	1.304	1.109	1.043	0.997	<0.001
120	1.369	1.117	1.050	1.044	<0.001
150	1.329	1.070	1.041	1.037	<0.001
P–Value^b^	<0.001	0.002	0.409	0.560	SEM = 0.0449^c^

^a^Temperature effect within day;

^b^Day effect over temperature;

^c^For n = 20.

On the other hand, no degradation of creatinine (P > 0.05) was observed in urine samples, demonstrating that dilution and correction of pH to values below three inhibit the degradation of the creatinine molecule. Wyss and Kaddurah-Daouk [8] reported that specific enzymes from microorganisms that can develop in urine degrade creatinine to acetic acid and methylguanidine under conditions of pH close to normal urine (8.4 to 8.7).

The urine that did not have added creatine showed an interaction between storage time and temperature (P < 0.05). From Days 1 to 15 of storage, there was no difference (P > 0.05) in the relative creatinine concentration in the samples. Thus, it is possible to infer that the diluted urine sample with adjusted pH values below three can be stored at any of the temperatures that were tested until fifteen days of storage.

However, between the 30th and 150th days of storage, there was an effect of storage time and/or temperature on the creatinine concentration (P < 0.05). Urine stored at room temperature and at 4 °C was influenced (P < 0.05) by storage time, with an increase in relative creatinine concentration (Fig 2). These data agree with Van Niekerk et al. [11], who, when evaluating the recovery of creatinine in urine samples from sheep, found an increase in the urinary concentration of creatinine when it was stored acidified (pH 2.5 to 3.5) at a temperature between 28 and 39 °C for 150 days.

On Day 30, the creatinine concentration increased approximately 17% relative to the creatinine observed on day zero. On Day 150, the creatinine concentration was approximately 33% higher than the reference value. These increases lead to an overestimation of creatinine concentration in urine, which may lead to experimental errors when using creatinine as an indicator of urinary volume. This allows us to infer that some compound present in the urine is being transformed into creatinine.

This increase in relative creatinine concentrations in the cattle’s urine may be linked to the excretion of creatine in the urine, since the balance of the creatinine molecule (creatine ↔ creatinine) in vitro is largely dependent on temperature and pH, where creatine is favored at basic pH and low temperature; furthermore, creatinine has an increase in its concentration when there are high temperatures and acidic environments [11, 12]. However, more experiments on this subject are necessary.

It should be noted that the concentrations of added creatine are much higher than those that could possibly be found in the urine of cattle, since the excretion of creatine in the urine is low. Data related to creatine excretion in cattle urine were not found. In humans, creatine excretion in urine is considered low. Vici et al. [13] evaluated the supply of supplements such as ornithine, lysine, arginine, citrulline and creatine to human patients who had hyperammonemia and observed concentrations of creatine below 11 micromole per kilo (μmol/kg) of body weight. These same authors, when citing Pavry et al., described the reference values for creatine excretion in urine to be 41 to 104 μmol/kg of body weight, not being influenced by age or sex [14].

Although the variation demonstrated was less than that shown at room temperature, the samples stored under refrigeration did show significant variation over the storage time (P < 0.05); this outcome suggests that even under refrigeration, it is possible to have increases in creatinine recovery.

The samples stored at temperatures that promoted urine freezing (-20 °C and -40 °C) were not influenced (P > 0.05) by time or temperature (Table 1), demonstrating that they are the best ways of storing urine for times longer than 15 days.

## 4. Conclusion

Cattle urine samples can be stored at any temperature for up to fifteen days after collection to determine the creatinine concentration. Samples that need storage times longer than fifteen days must be frozen at -20 °C or -40 °C to avoid creatinine concentration changes.

## References

[1] Chen XB and Ørskov ER. Research on urinary excretion of purine derivatives in ruminants: past, present and future. In: Makkar HPS, and Chen XB (eds) Estimation of Microbial Protein Supply in Ruminants Using Urinary Purine Derivatives. Springer, Dordrecht 2004.

[2] Chen XB and Gomes MJ. Estimation of microbial protein supply to sheep and cattle based on urinary excretion of purine derivates–on overview of technical details. Buscksburnd: Rowett Research Institute. International Feed Resources Unit, 1992, (Occasional publication), p. 21.

[3] Valadares RFD, Broderick SC, Valadares Filho SC, and Clayton MK. Effect of replacing alfafa silage with high misture on ruminal protein synthesis estimated from excretion of total purine derivatives. Journal of Dairy Science, 1999, 82, 2686–2696. doi: 10.3168/jds.S0022-0302(99)75525-6 10629816

[4] Rennó LN, Valadares RFD, Leão MI, Valadares Filho SC, Silva JFC, Cecon PR, et al. Microbial protein production obtained by the urinary purine derivatives in steers. Brazilian Journal of Animal Science, 2000, 29, 1223–1234.

[5] Silva Júnior JM, Rennó LN, Valadares Filho SC, Paulino MF, Detmann E, Menezes GCC, et al. Evaluation of collection days and times to estimate urinary excretion of purine derivatives and nitrogen compounds in grazing Nellore cattle. Livestock Science, 2018, 217, 85–97. doi: 10.1016/j.livsci.2018.09.016

[6] Silva Júnior JM, Rodrigues JPP, Valadares Filho SC, Detmann E, Paulino MF, and Rennó LN. Estimating purine derivatives and nitrogen compound excretion using total urine collection or spot urine samples in grazing heifers. Journal of Animal Physiology and Animal Nutrition, 2021, 105, 861–873. doi: 10.1111/jpn.13525 33704839

[7] Harper HA, Rodwell VW, and Mayes PA. Manual de química fisiológica. 5th ed. Atheneu, São Paulo, Brazil, 1982, p. 570.

[8] Wyss M and Kaddurah-Daouk R. Creatine and creatinine metabolism. Physiology Reviews, 2000, 80, 1107–1213. doi: 10.1152/physrev.2000.80.3.1107 10893433

[9] Ardalan M, Batista E, Armendariz C, and Titgemeyer E. Guaniclimoaceti acid as a precursor of creatine for cattle. Kansas Agricultural Experimental Station Research Reports, 2015, 1, 1–7. doi: 10.4148/2378-5977.1150

[10] Bloch K, Schoenheimer R, and Rittemberg D. Rate of formation and disappearance of body creatinine in normal animals. Journal of Biology Chemistry, 1941, 138, 155–166. doi: 10.1016/S0021-9258(18)51421-6

[11] Van Niekerk BDH, Bensadoun A, Paladines OL, and Reid JT. A study of the conditions affecting the rate of excretion and stability of creatinine in sheep urine. Journal of Nutrition, 1963, 79, 373–380. doi: 10.1093/jn/79.3.373 14010448

[12] Lempert C. The chemistry of the glycocyamidines. Chemistry Reviews, 1959, 59, 667–736. doi: 10.1021/cr50028a005

[13] Vici CD, Bachamann C, Gambarara M, Colombo JP, and Sabetta G. Hyperornithinemia-Hyperammonemia-Homocitrullinuria syndrome: Low creatine excretion and effects of citrulline, arginine, or ornithine supplement. Pediatrician Research, 1987, 22, 364–367. doi: 10.1203/00006450-198709000-00025 3116497

[14] Forbes GB and Bruining GJ. Urinary creatinine excretion and lean body mass. American Journal of Clinical Nutrition, 1978, 29, 1359–1366. doi: 10.1093/ajcn/29.12.1359 998546

